# Comparison of the Ultrasound Visibility of Tissue Markers in Metastatic Lymph Nodes after Neoadjuvant Chemotherapy in Patients with Breast Cancer

**DOI:** 10.3390/diagnostics12102424

**Published:** 2022-10-07

**Authors:** Ka Eun Kim, Eun Young Ko, Boo-Kyung Han, Eun Sook Ko, Ji Soo Choi, Haejung Kim, Jeong Eon Lee, Hyunwoo Lee

**Affiliations:** 1 Department of Radiology and Center for Imaging Science, Samsung Medical Center, Sungkyunkwan University School of Medicine, Seoul 06351, Korea; 2 Department of Surgery, Samsung Medical Center, Sungkyunkwan University School of Medicine, Seoul 06351, Korea; 3 Department of Pathology, Samsung Medical Center, Sungkyunkwan University School of Medicine, Seoul 06351, Korea

**Keywords:** breast cancer, clips, lymph nodes, axilla, neoadjuvant chemotherapy, ultrasonography

## Abstract

This study aimed to investigate the differences in ultrasound (US) visibility for the localization of clipped metastatic lymph nodes after neoadjuvant chemotherapy (NAC), according to tissue marker type. This single-center retrospective study included 59 consecutive patients with breast cancer who underwent tissue marker insertion for histologically proven metastatic axillary lymph nodes before NAC, between March 2020 and August 2021. Two breast tissue markers were used: UltraClip™ (*n* = 29) and UltraCor™ Twirl™ (*n* = 30). The US visibility of tissue markers after NAC and the successful excision rate of the clipped lymph nodes were compared between the two types of tissue markers. UltraCor™ Twirl™ showed better overall US visibility than UltraClip™ after NAC (86.7% vs. 72.4%), but the difference was statistically insignificant. In the absence of residual metastatic lymph nodes on US after NAC (*n* = 32), UltraCor™ Twirl™ showed significantly better US visibility (83.3%, 15/18) than UltraClip™ (42.9%, 6/14; *p* = 0.027). The marker type was not associated with the successful excision of the clipped lymph node. UltraCor™ Twirl™ showed better US visibility than UltraClip™ in the metastatic axillary lymph nodes after NAC in the absence of residual suspicious lymph nodes on US.

## 1. Introduction

Neoadjuvant chemotherapy (NAC) was initially used in breast cancer patients with inoperable and locally advanced disease [1]. However, owing to the advantages of NAC, it is the treatment of choice for patients with unresectable, inflammatory breast cancer and locally advanced breast cancer that may be operable after NAC [2]. Additionally, NAC may be recommended to reduce the tumor burden and allow for breast-conserving and less-invasive axillary surgery [2]. The role of NAC has expanded over time to include patients with operable breast cancer and NAC followed by surgery for patients with clinically node-positive breast cancer is now widely used [3,4]. NAC resulted in the pathologic complete response (pCR) of axillary lymph node metastasis in approximately 40–75% of patients [3,5,6,7,8]. In patients expected to achieve axillary pCR after NAC, sentinel lymph node biopsy (SLNB) has become a better choice for directing axillary lymph node dissection (ALND) to prevent morbidity and complications such as lymphedema [9]. Several clinical trials have evaluated SLNB for determining the axillary nodal status after NAC, including the American College of Surgeons Oncology Group (ACOSOG) Z1071 and SENTINA (sentinel lymph node biopsy in patients with breast cancer before and after neoadjuvant chemotherapy) trials [8,10]. However, the false-negative rates (FNRs) of the two studies ranged from 12.6% to 14.2% [8,10].

To overcome the high FNRs of SLNBs and improve the accuracy of nodal assessment after NAC, targeted axillary dissection (TAD) was established. TAD involves the removal of the sentinel lymph node and the previously biopsy-confirmed metastatic lymph node through localization [11]. For TAD, tissue marker insertion for histologically confirmed metastatic lymph nodes is required prior to the initiation of NAC. Various methods of preoperative localization for clipped lymph nodes—including iodine-125 radioactive seeds, ultrasound (US)-guided wire localization, and tattooing—are performed after NAC. Unlike breast masses, mammography-guided localization is not feasible for axillary lymph nodes. Therefore, regardless of the type of preoperative localization, the US visibility of previously inserted tissue markers is important to achieve a successful localization.

However, US-guided localization of axillary lymph nodes after a partial or complete radiologic response to neoadjuvant chemotherapy may often be challenging to radiologists [12,13]. A few studies have reported the US visibility of tissue markers for axillary lymph node localization in animal model phantoms or in a small number of patients with different types of tissue markers [14,15]. However, to the best of our knowledge, no study has evaluated the visibility of tissue markers in metastatic axillary lymph nodes after showing a pCR on US. Therefore, we aimed to investigate the differences in the US visibility of tissue markers according to the tissue marker type in metastatic axillary lymph nodes with or without residual suspicious lymph nodes after NAC.

## 2. Materials and Methods

This retrospective study was approved by the Institutional Review Board in Samsung Medical Center (IRB No. 2022-04-099), and the requirement for obtaining an informed consent was waived because of the retrospective study design.

### 2.1. Patients

From March 2020 to August 2021, we inserted tissue markers for histologically proven metastatic axillary lymph nodes in patients with breast cancer scheduled for NAC. Tissue marker insertion in axillary lymph nodes was mainly performed in patients who were expected to have a high possibility of undergoing axillary pCR, including those with human epidermal growth factor receptor 2 (HER2)-positive or triple-negative breast cancer.

**Figure 1:**
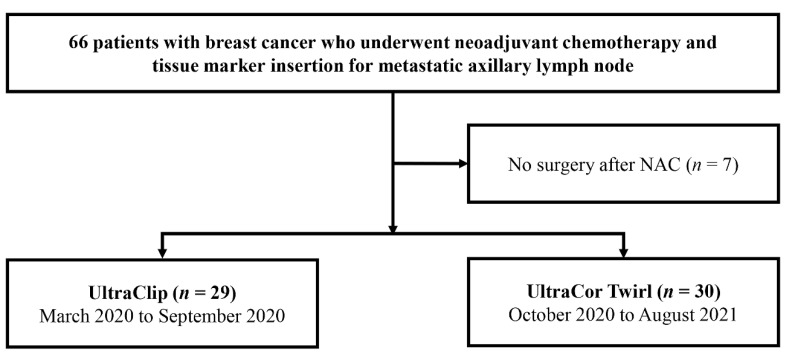
Flowchart of the participant selection process.

We retrospectively reviewed the medical records of all included patients and images of the inserted tissue markers. Seven patients who did not undergo breast cancer surgery after NAC were excluded. Finally, 59 consecutive patients—including 29 with UltraClip™ and 30 with UltraCor™ Twirl™—were included in the final analysis (Figure 1).

All patients underwent initial breast US, including that of the bilateral axilla, for nodal staging. Histological confirmation of suspicious axillary lymph nodes was performed via fine-needle aspiration or core needle biopsy.

### 2.2. Tissue Marker Insertion for Axillary Lymph Nodes before NAC

We used radiopaque tissue markers with a special coating for US visibility. In addition, the tissue markers were composed of MRI-compatible metals and had reduced metal artifacts. After histologic confirmation, we re-identified the metastatic lymph nodes that were biopsied before and inserted the tissue marker into the cortex of the metastatic lymph nodes prior the initiation of NAC. Tissue marker insertion was performed by one of seven breast radiologists with 2–27 years of experience in breast imaging. Two types of tissue markers were used: (1) the UltraClip™ dual-trigger breast tissue marker, ultrasound-enhanced titanium (BARD^®^, Tempe, AZ, USA) and (2) UltraCor™ Twirl™ Breast Tissue Marker (BARD^®^, Tempe, AZ, USA). UltraClip is a ribbon-shaped single-strain tissue marker, whereas UltraCor Twirl is a ring-shaped coiled tissue marker. From March 2020 to September 2020, UltraClip™ markers were inserted, and from October 2020 to August 2021, UltraCor™ Twirl™ markers were inserted. The US-guided tissue marker insertion was performed using one of three US devices: an IU22 (Philips Advanced Technology Laboratories, Bothell, DC, USA), Aixplorer US (SuperSonic Imagine, Aix-en-Provence, France), or RS80A (Samsung Medison Co., Ltd., Seoul, Korea) with linear array transducers. After insertion, the location of the tissue marker was confirmed on US immediately after implantation, and the axillary view of the mammography was also used to confirm the location of the tissue marker.

### 2.3. Localization of Tissue Marker-Inserted Axillary Lymph Nodes after NAC

After NAC, preoperative US-guided localization of the tissue marker-inserted axillary lymph nodes was performed on the day of surgery. This was performed by one of the breast radiologists who inserted the tissue markers. Wire (*n* = 27) and tattooing (*n* = 17) localizations were performed in 44 patients. In wire localization, the axillary view of the post-localization mammography was obtained to confirm the location of the wire and the tissue marker. For tattooing localization, 1 mL of Charcotrace™ Black ink (Phebra, Lane Cove West, Australia) was injected at the location of the tissue marker.

For localization, we reviewed the previous US images taken at the time of tissue marker insertion. The position and degree of arm elevation were set to be the same as those for tissue marker insertion. When a tissue marker was not identified on US, localization was performed at the presumed clip insertion site by measuring the distance from the skin and considering the surrounding structures.

### 2.4. Pathological Correlation after the Surgery

In all patients, the clipped lymph nodes were surgically excised in addition to SLNB. Based on the frozen section histologic results of the TAD, an additional ALND was performed in some patients. Specimen mammography of the nodal specimen was carried out to confirm the successful retrieval of tissue marker-inserted lymph nodes. The pathologists indicated the presence of a clip or tattoo within the lymph node in the pathology report, whether it was a sentinel lymph node, and the presence of residual metastasis.

### 2.5. Data Analysis

We retrospectively reviewed patients’ clinical records, including age, tumor stage (American Joint Committee on Cancer prognostic stage 8th edition), tumor subtype, and type of surgery. The US visibility of tissue markers and the presence of residual suspicious lymph nodes on US were determined based on the radiology reports provided by the radiologists who performed the localization on the day of surgery. The tissue marker type inserted into the lymph node, whether preoperative localization was performed, the method of localization, whether specimen mammography was performed, and the successful excision rate of the tissue marker-inserted lymph nodes were evaluated.

The patients were divided into two groups according to the tissue marker type: UltraClip and UltraCor Twirl. The US visibility of tissue markers within the metastatic lymph nodes after NAC was compared between the two groups. Moreover, the US visibility of tissue markers in patients with no residual suspicious lymph node after NAC was compared. The successful excision rate of the clipped lymph nodes was compared between the groups. The relationship between the successful excision rate of the clipped lymph nodes and the tissue marker type was also analyzed. The characteristics of patients between the two groups were also compared. Fisher’s exact test, independent t-test, and chi-square test were used for statistical analyses. A two-sided *p*-value of <0.05 was considered significant for all statistical tests. Statistical analyses were performed using SPSS software (version 27.0, Chicago, IL, USA).

## 3. Results

The mean age of the 59 patients who had the tissue marker inserted in the metastatic axillary lymph nodes was 49.2 ± 10.3 years. No significant difference was observed in the patient characteristics between the two groups of different tissue markers (all *p* > 0.05) (Table 1). Among the 59 patients, 32 (32/59, 54.2%) underwent breast-conserving surgery after NAC and 34 (32/59, 54.2%) underwent TAD without further axillary dissection. Among the patients, 44 (44/59, 74.6%) underwent US-guided localization of the clipped lymph node before surgery—whereas 15 (15/59, 25.4%) underwent axillary surgery without localization.

### 3.1. Comparison of the US Visibility between the Two Tissue Markers after NAC

**Figure 2:**
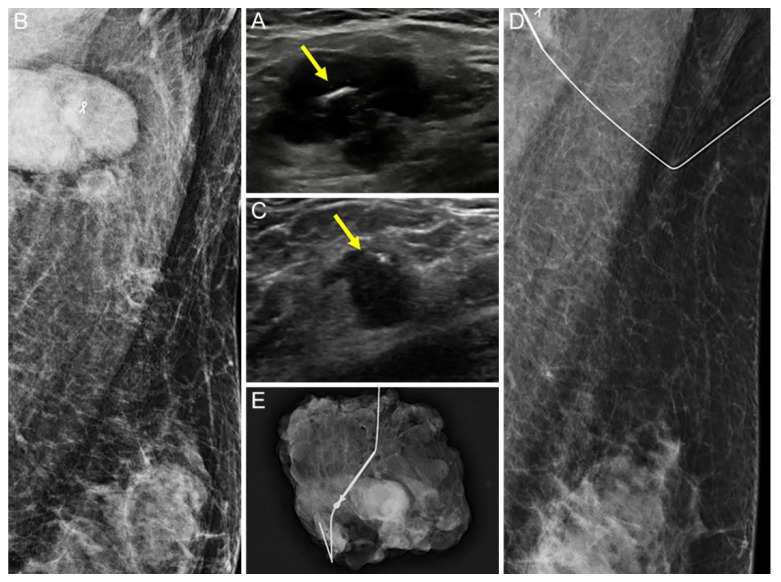
A 51-year-old woman with left breast cancer and ipsilateral axillary metastatic lymph node (LN) underwent UltraClip insertion before neoadjuvant chemotherapy (NAC). (**A**,**B**): The clip (arrow) was deployed at the cortex of the histologically proven metastatic LN by ultrasound (US) (**A**), and the location was confirmed by mammography (**B**). (**C**–**E**): After NAC, the size of the LN decreased, but remained suspicious on US. The clip (arrow) was also easily identifiable in the residual metastatic LN on US (**C**): US-guided wire localization was performed, and the location was confirmed by mammography (**D**). The wire was successfully located at the clipped LN, and subsequent specimen mammography confirmed that it was successfully retrieved (**E**).

**Figure 3:**
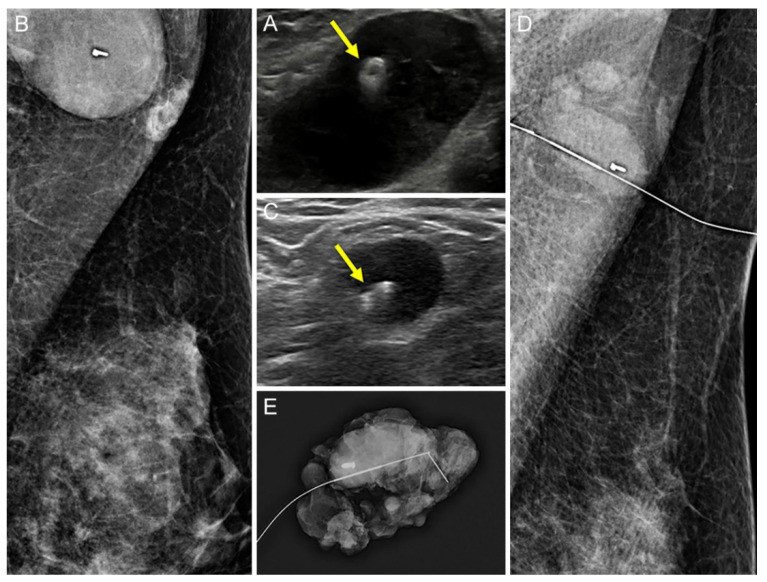
A 37-year-old woman with left breast cancer and ipsilateral axillary metastatic lymph node (LN) underwent UltraCor Twirl insertion before neoadjuvant chemotherapy (NAC). (**A**,**B**): The clip (arrow) was deployed at the cortex of the histologically proven metastatic LN by ultrasound (US) (**A**), and the location was confirmed by mammography (**B**). (**C**–**E**): After NAC, the size of the LN decreased, although it remained suspicious on US. The clip (arrow) was also easily identifiable in residual metastatic LN on US (**C**): US-guided wire localization was performed and confirmed by mammography (**D**). The wire was successfully located at the clipped LN and subsequent specimen mammography confirmed the successful retrieval (**E**).

**Figure 4:**
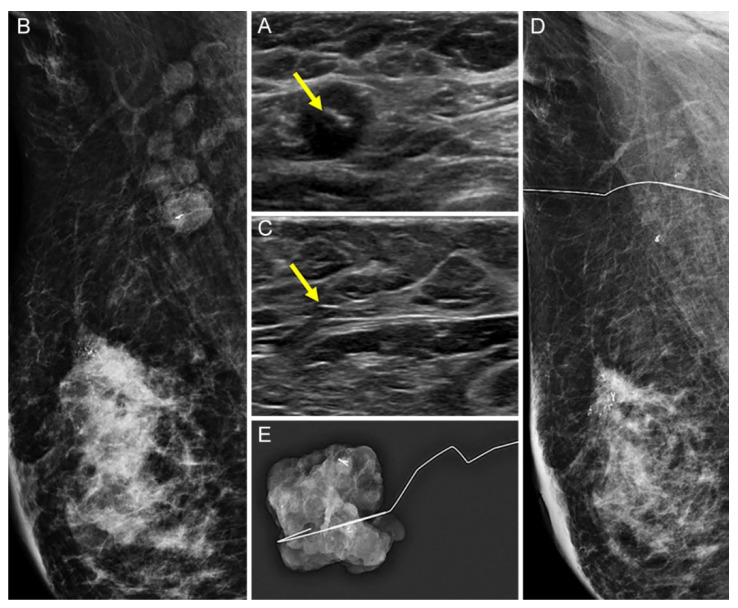
A 30-year-old woman with right breast cancer and ipsilateral axillary metastatic lymph node (LN) underwent UltraClip insertion prior to neoadjuvant chemotherapy (NAC). (**A**,**B**): The clip (arrow) was deployed at the cortex of the histologically proven metastatic LN by US (**A**) and the location was confirmed by mammography (**B**). (**C**–**E**): After NAC, no residual suspicious lymph node was detected on US and the clip location was not clear. An echogenic linear structure, suggesting the presence of the metal clip (arrow), was found at the presumed area of the previously clipped LN. A US-guided wire localization for the echogenic line considered a clip was performed (**C**): On mammography, however, the wire was not correctly located at the clipped LN (**D**). Subsequent specimen mammography confirmed the successful retrieval of the clipped LN (**E**).

**Figure 5:**
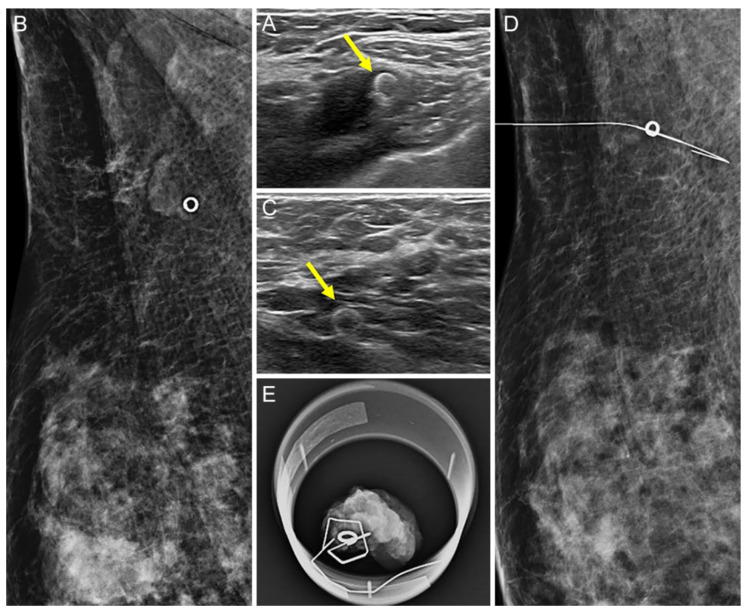
A 44-year-old woman with right breast cancer and ipsilateral axillary metastatic lymph node (LN) underwent UltraCor Twirl insertion prior to neoadjuvant chemotherapy (NAC). (**A**,**B**): The clip (arrow) was deployed at the cortex of the histologically proven metastatic LN by US (**A**) and the location was confirmed by mammography (**B**). (**C**–**E**): After NAC, residual suspicious lymph nodes were no longer detected on US, but the previously inserted ring-shaped clip (arrow) was clearly visible (**C**). US-guided wire localization of the visible clip was performed, and the wire was successfully located at the clipped LN on mammography (**D**). Subsequent specimen mammography confirmed the successful retrieval of the clipped LN (**E**).

The US visibility of tissue markers was better in the UltraCor Twirl group (26/30, 86.7%) than in the UltraClip group (21/29, 72.4%), but the difference was statistically insignificant (*p* = 0.209; Figure 2 and Figure 3). However, in patients with no residual suspicious lymph nodes on US after NAC (*n* = 32), the tissue marker visibility was significantly better when using UltraCor Twirl (15/18, 83.3%, *p* = 0.027) than when using UltraClip (6/14, 42.9%) (Table 2; Figure 4 and Figure 5).

### 3.2. Comparison of the Successful Excision Rates of the Clipped Lymph Nodes between the Two Tissue Markers

The successful excision rates were evaluated—with the exception of one patient in whom it was uncertain whether the clipped lymph node had been surgically removed because there was no specimen mammography of axillary lymph nodes and no description of the clip in the pathologic report after surgery. The tissue marker-inserted lymph nodes were successfully removed in 51 patients (51/58, 87.9%), including 26 patients in the UltraClip group (26/29, 89.7%) and 25 patients in the UltraCor Twirl group (25/29, 86.2%); however, the removal failed in seven patients (7/58, 12.1%).

Regardless of the tissue marker type, the clipped lymph node was successfully excised in 95.3% (41/43) of the patients who underwent preoperative US-guided localization and in 66.7% (10/15) of the patients who did not undergo preoperative localization. Patients who underwent preoperative US-guided localization for the clipped lymph nodes showed a higher successful excision rate than those without localization (*p* = 0.010). Meanwhile, the tissue marker type was not associated with the successful excision of the clipped lymph nodes (*p* = 1.000; Table 3).

In patients who underwent preoperative localization, the tissue marker type was not associated with the successful excision rate of the clipped lymph nodes (*p* = 0.233). Both types of tissue markers showed excellent success rates (UltraClip, 100% (22/22) and UltraCor Twirl, 90.5% (19/21)).

## 4. Discussion

Radiologists often experience difficulties in US-guided localization of clipped lymph nodes after NAC because residual axillary lymph nodes and tissue markers are not clearly visible on US owing to the high rate of pCR in patients with breast cancer. In our study, 34.4% (11/32) of the tissue markers in the axilla were no longer detectable on US after NAC in the absence of residual suspicious lymph nodes on US. In Koo et al.’s evaluation of the visibility of breast tissue markers, approximately 28.6% of tissue markers were not seen on US when the residual tumors were no longer detectable on US [16]. Likewise, the localization of tissue markers without a surrounding mass or cortical thickening is more challenging in the axilla than in the breast tissue. The axilla contains multiple hyperechoic tissue interfaces and commonly shows a heterogeneous echotexture on US, which mimics a hyperechoic metallic clip. Unlike the hypoechoic fibrotic changes of breast masses after NAC, the shrunken axillary lymph nodes after NAC do not show significant fibrosis of the adjacent tissue, making it difficult to identify the tissue markers. Unlike breast masses, there are no established methods for describing the distance and direction of the axillary lymph nodes.

Thus, a clear US visibility of tissue markers in the axilla is important for the successful localization of clipped lymph nodes after NAC. This enables radiologists to reduce the time and effort required for localization and to increase the accuracy of localizing these lymph nodes. In our study, the UltraCor Twirl showed better overall US visibility after NAC than UltraClip, although the difference was statistically insignificant. However, in patients with no residual metastatic lymph nodes on US, UltraCor Twirl showed significantly better US visibility than UltraClip. If a thick cortex of lymph nodes is noted around the tissue marker, it is likely to be visible on US, regardless of the tissue marker type. However, in the absence of a suspicious lymph node on US, the US visibility of the tissue marker itself becomes a more important factor in ensuring successful localization.

Portnow et al. [15] reported the US visibility of four types of tissue markers—including UltraCor Twirl and UltraClip—in animal tissue models stimulating axillary echotexture, and UltraCor Twirl demonstrated the highest score on the grading system used by radiologists. Lim et al. [14] reported that UltraCor Twirl showed the best US visibility and highest excision rate after NAC among the four different types of tissue markers, including UltraCor Twirl and UltraClip, in 14 patients. Our study results support those of previous studies; however, we investigated a larger number of patients and analyzed the visibility in the absence of residual suspicious lymph nodes on US after NAC.

The superior US visibility of the UltraCor Twirl in the axilla may be related to the shape and surface area of the tissue markers. The larger surface area of the coiled tissue marker with an increased interface with the surrounding tissue allows better reflection of sound wave energy back to the transducer—thus resulting in better visualization in a heterogeneous axillary echotexture. In addition, the ring-shaped tissue markers may be more prominent and exaggerated in the background linear echogenic strands of the axillary tissue than the ribbon-shaped tissue marker, which is often seen on US as a linear echogenic line. No additional complications such as bleeding or migration occurred and no difference was found in the MRI metal artifacts with UltraCor Twirl in our study. If the clipped lymph nodes are not successfully localized, surgeons might need to spend more time and put more effort in to retrieve clipped lymph nodes.

Therefore, the better US visibility of tissue markers may reduce the time and effort required by surgeons and radiologists. However, different results may be obtained in a larger study population, since this was a single-institution study conducted in only 59 patients.

Our study has some limitations: First, this was a single-center retrospective study, although it included a larger sample size compared with that of previous studies on the US visibility of clipped lymph nodes. Second, the US visibility of tissue markers was determined by one of seven radiologists with various degrees of experience in localizing a clipped lymph node after NAC. Therefore, an inter-observer variability may have been present. Third, we assumed that better US visibility might reduce the time and effort of radiologists; however, we did not measure the time required for the localization of clipped lymph nodes.

In conclusion, the tissue marker type inserted in the axillary lymph nodes affected US visibility in the absence of residual lymph nodes on US after NAC. A tissue marker such as coiled ring shape rather than linear shape, which is more visible, is recommended for axillary lymph nodes in patients scheduled for NAC.

## Figures and Tables

**Table 1:** Patient characteristics.

	Total (*n* = 59)	UltraClip (*n* = 29)	UltraCor Twirl (*n* = 30)	*p*-Value
Age ^a^, years, mean (SD)	49.2 (10.3)	49.3 (10.1)	49.0 (10.6)	0.899 ^b^
Tumor stage, n (%)				0.087 ^c^
IB	16 (27.1)	3 (10.3)	13 (43.3)	
IIA	8 (13.6)	4 (13.8)	4 (13.3)	
IIB	11 (18.6)	7 (24.1)	4 (13.3)	
IIIA	11 (18.6)	6 (20.7)	5 (16.7)	
IIIB	5 (8.5)	4 (13.8)	1 (3.3)	
IIIC	8 (13.6)	5 (17.2)	3 (10.0)	
Tumor subtype, n (%)				0.328 ^d^
ER	23 (39.0)	9 (31.0)	13 (46.7)	
Her-2	21 (35.6)	13 (44.8)	8 (26.7)	
TNBC	15 (25.4)	7 (24.1)	8 (26.7)	
Breast surgery, n (%)				0.235 ^d^
BCS	32 (54.2)	18 (62.1)	14 (46.7)	
TM	27 (45.8)	11 (37.9)	16 (53.3)	
Axillary surgery, n (%)				0.228 ^d^
TAD	34 (57.6)	19 (65.5)	15 (50.0)	
TAD with ALND	25 (42.4)	10 (34.5)	15 (50.0)	
Localization, n (%)				0.824 ^d^
Yes	44 (74.6)	22 (75.9)	22 (73.3)	
No	15 (25.4)	7 (24.1)	8 (26.7)	

SD: standard deviation; HER2: human epidermal growth factor receptor 2; BCS: breast-conserving surgery; TM: total mastectomy; TAD: targeted axillary dissection; ALND: axillary lymph node dissection. ^a^ Shapiro–Wilk test was performed for age. ^b^ Independent *t*-test was performed. ^c^ Fisher’s exact test was performed. ^d^ Chi-square test was performed.

**Table 2:** Comparison of US visibility between the two tissue markers.

	UltraClip		UltraCor Twirl		*p*-Value
US Visibility	Yes	No	Yes	No	
All cases (*n* = 59)	29		30		0.209 ^a^
No. (%)	21 (72.4)	8 (27.6)	26 (86.7)	4 (13.3)	
No residual LN on US (*n* = 32)	14		18		0.027 ^a^
No. (%)	6 (42.9)	8 (57.1)	15 (83.3)	3 (16.7)	

LN: lymph node. ^a^ Chi-square test was used.

**Table 3:** Successful excision rates between the two tissue markers.

	UltraClip		UltraCor Twirl		*p*-Value
Successful Excision	Yes	No	Yes	No	
All cases (*n* = 58)	29		29		1.000 ^a^
No. (%)	26 (89.7)	3 (10.3)	25 (86.2)	4 (13.8)	
With localization (*n* = 43)	22		21		0.233 ^a^
No. (%)	22 (100.0)	0 (0)	19 (90.5)	3 (14.3)	

^a^ Fisher’s exact test was used.

## Data Availability

The data are not available for public access because of patient privacy concerns but are available from the corresponding author on reasonable request.
